# Dietary Influence on Drug Efficacy: A Comprehensive Review of Ketogenic Diet–Pharmacotherapy Interactions

**DOI:** 10.3390/nu16081213

**Published:** 2024-04-19

**Authors:** Simona Cristina (Nicolescu) Marinescu, Miruna-Maria Apetroaei, Marina Ionela (Ilie) Nedea, Andreea Letiția Arsene, Bruno Ștefan Velescu, Sorina Hîncu, Emilia Stancu, Anca Lucia Pop, Doina Drăgănescu, Denisa Ioana Udeanu

**Affiliations:** 1 Faculty of Pharmacy, Carol Davila University of Medicine and Pharmacy, 6, Traian Vuia Street, 020956 Bucharest, Romaniaandreea.arsene@umfcd.ro (A.L.A.); bruno.velescu@umfcd.ro (B.Ș.V.); sorina.calugaru@drd.umfcd.ro (S.H.); emilia.stancu@umfcd.ro (E.S.); anca.pop@umfcd.ro (A.L.P.); doina.draganescu@umfcd.ro (D.D.); denisa.udeanu@umfcd.ro (D.I.U.); 2 Amethyst Radiotherapy Center, 42, Drumul Odăi, 075100 Otopeni, Romania; 3 Marius Nasta Institute of Pneumophthiology, 90, Viilor Street, 050159 Bucharest, Romania; 4 Fundeni Clinical Institute, 258, Fundeni Street, 022328 Bucharest, Romania

**Keywords:** ketogenic diet, nutritional ketosis, pharmacotherapeutics, drug interactions, chronic disease management

## Abstract

It is widely acknowledged that the ketogenic diet (KD) has positive physiological effects as well as therapeutic benefits, particularly in the treatment of chronic diseases. Maintaining nutritional ketosis is of utmost importance in the KD, as it provides numerous health advantages such as an enhanced lipid profile, heightened insulin sensitivity, decreased blood glucose levels, and the modulation of diverse neurotransmitters. Nevertheless, the integration of the KD with pharmacotherapeutic regimens necessitates careful consideration. Due to changes in their absorption, distribution, metabolism, or elimination, the KD can impact the pharmacokinetics of various medications, including anti-diabetic, anti-epileptic, and cardiovascular drugs. Furthermore, the KD, which is characterised by the intake of meals rich in fats, has the potential to impact the pharmacokinetics of specific medications with high lipophilicity, hence enhancing their absorption and bioavailability. However, the pharmacodynamic aspects of the KD, in conjunction with various pharmaceutical interventions, can provide either advantageous or detrimental synergistic outcomes. Therefore, it is important to consider the pharmacokinetic and pharmacodynamic interactions that may arise between the KD and various drugs. This assessment is essential not only for ensuring patients’ compliance with treatment but also for optimising the overall therapeutic outcome, particularly by mitigating adverse reactions. This highlights the significance and necessity of tailoring pharmacological and dietetic therapies in order to enhance the effectiveness and safety of this comprehensive approach to managing chronic diseases.

## 1. Introduction

As non-communicable diseases continue to impose a disproportionate burden, particularly in low- and middle-income countries with over 31.4 million deaths annually [1], the role of dietary interventions alongside pharmacological treatments gains prominence [2]. The ketogenic diet (KD), in particular, offers a novel approach to combating the rising tide of chronic diseases, including cardiovascular diseases, cancer, chronic respiratory diseases, and diabetes, by potentially mitigating the risk factors associated with these conditions [3].

The KD involves consuming less than 30 grammes of carbohydrates *per* day and maintaining a protein intake of 1.2–1.5 grammes *per* kilogramme of ideal body weight or 1.0–1.2 grammes *per* kilogramme of fat-free mass [4]. In a standard macronutrient distribution, lipids account for around 55% to 60%, protein for 30% to 35%, and carbs for 5% to 10%. The recommended daily carbohydrate limit for a 2000-calorie regimen, for example, would range between 20 and 50 grammes [5].

This diet generates ketosis by decreasing dietary carbs and increasing protein and fat intake, causing the body to consume fat as its primary source of energy instead of carbohydrates. The main goal of the KD is to reduce fat stores and enhance metabolic function [6]. There are four types of KDs: the long-chain triglyceride KD (LCT KD), the medium-chain triglyceride KD (MCT KD), the low-glycaemic index KD (LGI KD), and the modified Atkins KD [2].

Changes in lipid metabolism are notable when following the KD, with a common focus on its effects on blood lipids. During a shortage of glucose, the energy focus switches from glycolysis to the breakdown of fatty acids [7]. Despite being high in fat, the KD is able to surprisingly reduce heart disease risk factors, as proven by studies indicating decreased total cholesterol, increased HDL, and reduced triglyceride (TG) amounts [8]. The diet’s focus on reducing carbohydrates helps lower insulin levels, improve insulin sensitivity, and increase fat breakdown, leading to a decrease in blood lipids [9]. In particular, the KD alters metabolism to promote higher levels of lipid oxidation along with liver ketogenesis, leading to a decrease in liver fat [10,11,12]. Furthermore, it enhances fibroblast growth factor-1, which helps in TG clearance [13] and could potentially modify the dimension of LDL-cholesterol fragments to lower the risk of cardiovascular disease. Moreover, reducing dietary carbohydrates through the KD hinders the production of cholesterol, which is influenced by insulin and plasma glucose levels [14].

The KD has a substantial effect on glucose, resulting in positive aspects consisting of enhanced glucose tolerance and increased sensitivity to insulin. When following a KD, the body shifts to burning fat for energy when carbohydrates are restricted. This results in the generation of ketone bodies within a specific range known as nutritional ketosis (0.5–3.0 mmol/L) while also lowering circulating glucose levels without impacting blood pH [15,16,17]. This reduces the absorption of monosaccharides in the intestines, leading to a decrease in plasma glucose levels [18]. Moreover, the KD enhances insulin sensitivity and attenuates HOMA-IR scores [15,19,20,21].

**Figure 1:**
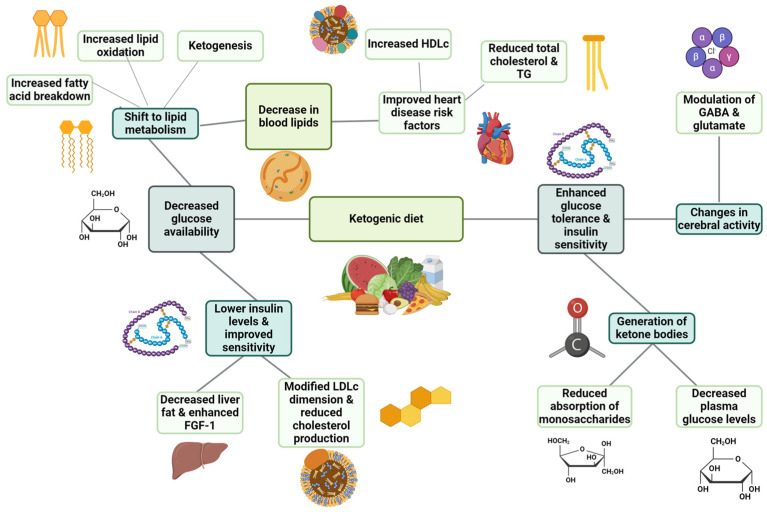
KD metabolic effects (created with BioRender.com) (accessed on 13 March 2024).

Moreover, the KD changes the cerebral activity and the management of seizures by involving the neurological chemicals GABA and glutamate, during which ketone bodies reduce the utilisation of glucose and affect neurotransmitter functions [22,23]. By modifying glutamate and GABA levels, the diet plays a key role in its antiepileptic effects. This is achieved by restricting glutamate decarboxylase and boosting GABA production via metabolic processes [24,25,26]. Higher levels of GABA have been found in various studies, both in murine models and clinical settings. These elevated levels are associated with elevated levels of β-hydroxybutyrate, which aid in reducing neuronal activity and improving seizure management. Furthermore, KB affects ion channels and neurotransmitter carriers, impacting the glutamate-to-ketone percentage and potentially decreasing epileptic events [27,28]. Figure 1 summarises the most important health benefits of the KD.

When it comes to cancer cases, the KD has been shown to decrease inflammation by lowering TNF-α expression, promoting IL-10 expression, reducing the NLRP3 inflammasome, and decreasing the amount of glucose utilised by tumour cells. These processes result in reduced tumour growth, improved cancer survival rates, and increased effectiveness of chemotherapy and radiotherapy [29]. The KD has shown promising results as an additional treatment for gliomas [30,31]. In this scenario, drug interactions may arise in patients diagnosed with cerebral tumours who are also receiving pharmacologic treatment for other conditions.

Although highly acknowledged for its beneficial effects on health and in managing chronic conditions, the KD can cause severe health problems if followed without surveillance. A diet that is severely restricted in carbohydrates often excludes or drastically reduces the intake of vegetables, fruits, and whole cereals while substantially raising the consumption of animal-derived foods. Consequently, individuals following low-carbohydrate diets are susceptible to serious nutritional deficiencies [32,33]. Furthermore, the initiation of a dietary regimen rich in saturated lipids elevates the likelihood of developing coronary heart disease, atherosclerosis, and stroke [34]. Additional concerns linked to the ketogenic diet encompass renal dysfunction and reduced bone density [35]. Continuous nutritional monitoring is necessary for KDs to ensure their efficacy and minimise the risk of both immediate and long-term negative consequences. According to the guidelines provided by the International Ketogenic Diet Study Group for the most effective clinical management, it is recommended that a comprehensive team consisting of neurologists, nutritionists, dietitians, and paediatricians closely oversee patients in order to optimise the therapeutic outcomes [36]. Furthermore, in instances where patients present with various comorbidities, particularly those with liver and kidney dysfunction, it is imperative to prescribe the KD under rigorous medical oversight, accompanied by strict clinical and laboratory surveillance. This surveillance should encompass the monitoring of beta-hydroxybutyrate levels and drug plasma concentrations [37].

In the face of the escalating prevalence of chronic diseases, the KD emerges as a multifaceted intervention with potential impacts on metabolic processes, glucose regulation, lipid management, and neurological functions. This review aims to assess the interactions between the KD and pharmacotherapeutic agents and to elucidate both synergistic benefits and potential adverse effects arising from the concurrent administration of the KD and medications across various chronic conditions.

## 2. Methods

The purpose of this narrative review was to examine the effects of the ketogenic diet on drug pharmacokinetics and pharmacodynamics, specifically in relation to medications used to treat a variety of chronic pathologies. The primary objective was to compile data from the scientific literature in order to generate a comprehensive list of potential positive or negative consequences, as well as clinical implications for practitioners.

The literature search methodology encompassed the process of identifying several keywords tailored to our objectives: “ketogenic diet”, “chronic diseases management”, “dietary interventions for disease management”, “nutritional ketosis”, “diabetes management”, “metformin and ketogenic diet”, “SGLT-2 inhibitors and ketogenic diet”, “cardiovascular diseases and ketogenic diet”, “cardiovascular drugs and ketogenic diet”, “hypertension management and ketogenic diet”, “beta-blocking agents and ketogenic diet”, “neurological effects of ketogenic diet”, “antiepileptic drugs and ketogenic diet”, “neurotransmitter modulation and ketogenic diet”, “CNS drugs and ketogenic diet”, “psychiatric disorders and ketogenic diet”, “cancer therapy and ketogenic diet”, “ketogenic diet in oncology”, “chemotherapy and ketogenic diet”, “radiation therapy and ketogenic diet”, “oncologic drugs and ketogenic diet”, “ketogenic diet on gut microbiota”, “ketogenic diet effects”, “probiotics and ketogenic diet”, “microbiota-modifying medications”, “gastrointestinal health and ketogenic diet”, “lipophilic drugs”, “drugs with increased absorption in lipids”, “ketosis-disrupting drugs”, and “carbohydrate content in medications”. A comprehensive search was conducted on many databases, including Scopus, Web of Science, Google Scholar, and PubMed, using the identified keywords. The search was limited to publications published up until 1 January 2024. Furthermore, subsequent to the identification of pertinent papers, an analysis was conducted on the references within such articles in order to identify further studies. The present narrative review encompasses a comprehensive analysis of several studies that investigate the effects of the ketogenic diet on the pharmacokinetics and/or pharmacodynamics of the drug. These studies cover a range of methodologies, including experimental studies, observational studies, clinical studies, reviews, and case reports. The exclusion criteria comprised research that did not directly evaluate the influence of KD on pharmaceuticals, publications written in languages other than English, or articles that solely examined the effects of the ketogenic diet on specific medical conditions. In light of the narrative characteristics of our review, the data were qualitatively synthesised and classified based on the framing disease, kind of interaction, and drug subclass.

## 3. KD versus Antidiabetic Drugs

The significant increase in diabetes cases, rising from 108 million people in 1980 to 422 million in 2014, and its impact on around 2 million deaths in 2019 underscore the major public health issue it poses, especially in low- and middle-income countries. The significant rise in numbers, along with a 3% rise in diabetes-related deaths from 2000 to 2019, highlights the urgent requirement for comprehensive management strategies [38]. Studying the combined effects of the KD and diabetes medication is an important research focus that aims to improve treatment results and reduce side effects.

### 3.1. Metformin

Metformin is a commonly prescribed medication that offers significant advantages for glucose regulation and managing diabetes-related diseases [39]. The main mechanism of action involves changing the cell’s energy metabolism. Metformin demonstrates its major glucose-lowering impact by reducing hepatic gluconeogenesis and counteracting the effects of glucagon. When mitochondrial complex I is inhibited, it leads to impaired cAMP and protein kinase A signalling in reaction to glucagon. While not essential for metformin’s ability to lower blood sugar, activating 5′-AMP-activated protein kinase enhances insulin sensitivity primarily through the regulation of lipid metabolism [40,41].

Additional research has delved into metformin’s ability to inhibit cancer growth and advancement in different forms [42,43,44]. Evidence from cellular and preclinical studies supports the drug’s potential for cancer treatment. These studies demonstrate antineoplastic effects and tumour growth inhibition by targeting mitochondrial OXPHOS [45] and downregulating mTOR signalling [46]. A recent study has demonstrated that fasting-induced hypoglycaemia combined with metformin can hinder tumour growth by affecting the PP2A-GSK3βMCL-1 axis, a pathway involved in glioblastoma [47]. During a recent phase I clinical trial, researchers explored the combined effects of the KD with metformin and radiotherapy on gliomas. Individuals were placed on a modified Atkins diet in addition to receiving radiation therapy and metformin. The research showed that increased serum ketone levels, linked to metabolic stress believed to improve radiation effectiveness, were strongly connected to dietary changes, the use of metformin, and reduced insulin levels. This led to a beneficial pharmacodynamic interaction among the regimens [48].

### 3.2. SGLT-2 Inhibitors

Sodium-glucose cotransporter-2 (SGLT-2) inhibitors, commonly referred to as gliflozins or flozins, are medications used extensively to treat type 2 diabetes (T2D) owing to their benefits in reducing glucose levels and improving cardiovascular and renal function [49]. These include empagliflozin, dapagliflozin, canagliflozin, sotagliflozin, and ertugliflozin, which are antihyperglycemic drugs that inhibit the reabsorption of glucose within the lumen, reducing the tubular limit for glucose and promoting the elimination of glucose in the form of urine [50]. Urinary tract infections and diabetic ketoacidosis (DKA) are the most prevalent adverse effects [51]. Canagliflozin is the most probable inducer of DKA, with Dapagliflozin and Empagliflozin having a lower likelihood of inducing it [52].

**Figure 2:**
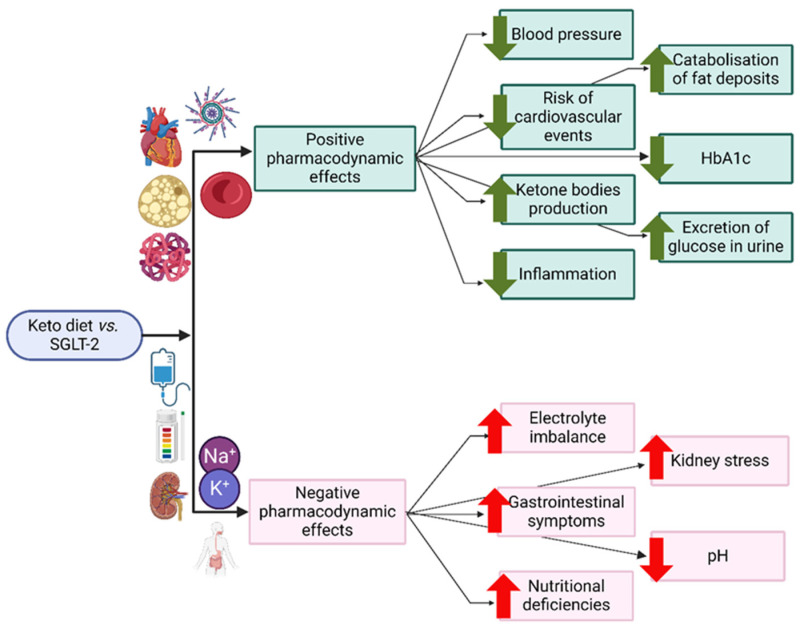
Keto and SGLT-2 interactions (adapted from [49]) (created with Biorender.com).

The KD and SGLT-2 inhibitors show great promise as treatment strategies for managing T2D and its associated problems. Recently, there has been a growing interest in both treatments because of their potential combined health benefits, including weight loss, enhanced insulin sensitivity, and reduced cardiovascular risk [49,53]. The positive and negative pharmacodynamic effects of the combination of KD and SGLT-2 are depicted in Figure 2.

## 4. KD versus Cardiovascular Drugs

According to the WHO, cardiovascular conditions, or CVDs, are the primary global contributing factor to mortality, resulting in 17.9 million deaths each year. Coronary heart disease, cerebrovascular accidents, and rheumatic heart disease are primarily triggered by strokes and cardiac events, with one-third happening prematurely in individuals under 70. Primary risk factors consist of inadequate eating habits, a sedentary lifestyle, tobacco consumption, and alcohol misuse [54].

The KD has been extensively explored as a therapeutic strategy for managing cardiovascular diseases. Research has linked the KD to a number of health benefits, including lower total cholesterol, higher HDL cholesterol, lower triglyceride levels, and lower LDL cholesterol [6,55,56,57]. There is evidence that a decrease in atherogenicity and an increase in the size and volume of LDL cholesterol particles are connected with the KD, which, in turn, reduces cardiovascular risk [58].

Yurista S.R. et al. suggested that ketone bodies produced by the KD may have various beneficial impacts on cardiovascular health. Ketone bodies can enhance endothelial function, reduce oxidative stress, enhance mitochondrial function, have anti-inflammatory effects, and alleviate cardiac remodelling. Additional systemic extracardiac effects may also have positive impacts on body mass index, blood sugar levels, and lipid composition and levels in patients with cardiovascular disease [59].

Various adverse consequences on heart health have been recorded with the use of the KD. Electrocardiographic evidence of QT prolongation and selenium insufficiency, both of which are linked to decreased cardiac function, are two side effects of the KD [60].

**Figure 3:**
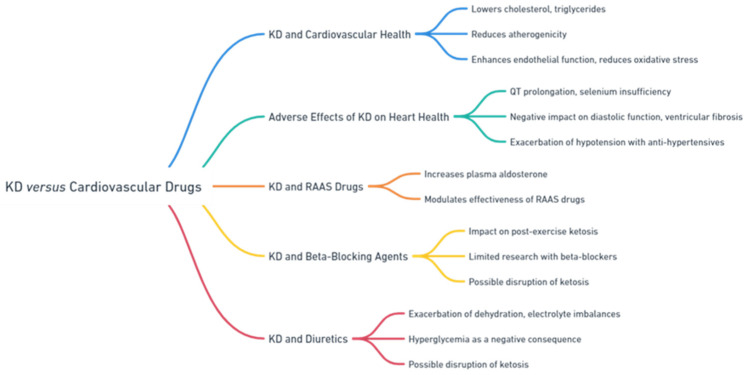
Overview of KD and cardiovascular pharmacotherapy.

Tao J. et al. conducted a study on the impact of a KD on diabetic cardiomyopathy, specifically examining cardiac function and underlying mechanisms. The study found that the KD has positive effects on metabolic indicators in diabetic mice, but it has negative impacts on heart diastolic function and leads to increased ventricular fibrosis. The study emphasised how ketone bodies impact T-regulatory cell activity, worsening heart problems through interactions with mitochondrial-associated endoplasmic reticulum membranes and the utilisation of fatty acids. It appears that despite the metabolic advantages, the KD has a predominantly adverse effect on cardiac remodelling in dilated cardiomyopathy, influenced by reduced T-reg cell function and increased fibroblast activation [61]. Figure 3 provides an extensive overview of the effects resulting from the concurrent administration of cardiovascular medications and KD.

### 4.1. Agents Acting on the Renin-Angiotensin System

The relationship between the KD and drugs acting on the renin–angiotensin–aldosterone system (RAAS) is a very promising research field, especially for dietary interventions and their endocrine effects. Recently, a study compared the action of KDs, with and without ketone salt supplements, to a low-fat diet (LFD) on the activity of RAAS in overweight and obese individuals. Their results identified that all dietary strategies had led to statistically significant weight loss. KDs increased plasma aldosterone without increasing levels of other key cardiometabolic risk factors adversely. This increase in aldosterone, with the direct association of the presence of ketone bodies, would strongly indicate a direct mechanistic link between the metabolic state of ketosis, induced by KDs, and an increase in aldosterone production. It is therefore implied that a physiological response to KDs modulating the effectiveness and side effects of drugs acting on the RAAS may be different from that evoked by LFDs [62].

This study investigates the effect of the low-protein diet supplemented with ketoacids (LPD+KA) on the RAAS in chronic kidney disease. It has been concluded that LPD+KA can reduce proteinuria and intrarenal activation of the RAAS. This effect proceeds independently of changes in renal hemodynamics and strongly implies the presence of a direct interaction of dietary components with the RAAS pathways. On the other hand, LPD+KA was shown to specifically reduce the expression of major RAAS components, such as angiotensin II and its receptor, in mesangial cells and the renal cortex. The mechanisms underlying these phenomena are considered to include the amelioration of nutritional metabolic disorders and oxidative stress [63]. This finding, therefore, is pertinent to the way in which dietary intervention, such as LPD+KA, can aid in increasing the efficacy of RAAS-blockading drug administration in CKD. Such combinations of diets with pharmacologic agents acting on the RAS system, therefore, would imply that they may have a synergistic effect and thus lead to possibly better clinical outcomes in CKD patients.

### 4.2. Beta-Blocking Agents

A study conducted 30 years ago examined the impact of a ketogenic diet when administered alongside beta-blockers. Exercise alone did not have an independent effect on post-exercise ketosis levels in carbohydrate-starved individuals. Additionally, the treatment with propranolol heightened ketosis levels in individuals who had exercised but reduced ketosis levels in those who had not exercised. Beta-blockers and exercise are both involved in mediating ketosis, with beta-blockers playing a more significant role than exercise. Glucose, insulin, and other metabolic indicators have minimal impact on this process, while the predominant changes are attributed to the impact of muscle metabolite flow on liver metabolism [64].

Currently, there are a limited number of studies examining the impact of beta-blockers when used in conjunction with KD. Research revealed that a low carbohydrate diet led to notable improvements in blood pressure levels [65]. An additional investigation examined the impact of a very-low-calorie ketogenic diet (VLCKD) on a group of female patients with obesity and hypertension. The study concluded that VLCKD is an effective therapeutic method for treating hypertension and obesity due to its positive metabolic and anti-inflammatory impacts. However, one exclusion criterion for the trial was women undergoing treatment with beta-blockers and/or other antihypertensive medications [66]. It has to be acknowledged that combining a KD with antihypertensive drugs may lead to a significant decrease in blood pressure, necessitating more research.

For patients with heart failure and coronary artery disease, beta-blockers are a crucial part of guideline-directed therapy. They are also commonly used to treat hypertension [67]. Non-selective beta-blockers have been widely recognised to be correlated with a deterioration in lipid and glycaemic control [68]. Beta-blockers can affect hypoglycaemia symptoms in insulin-dependent diabetes and increase the risk of hyperglycaemia in non-insulin-dependent diabetics. Beta-blockers have the ability to raise blood glucose levels and counteract the effects of oral hypoglycaemic medications [69]. Considering all of these factors and the fact that nutritional ketosis, in which ketone bodies serve as the body’s primary energy source, is the most important state to maintain on the KD [70], it is noteworthy that patients taking beta-blockers and attempting to adhere to a KD may find it considerably more challenging to maintain ketosis.

### 4.3. Diuretics

In accordance with the 2020 Global Practice Guidelines for Hypertension of the International Society of Hypertension, diuretics are critical first-line treatments not only for hypertension but also for hypertension in conjunction with other common comorbidities [71]. By increasing the elimination of water and electrolytes, they exert their hypotensive effect. Thus, electrolyte imbalances and dehydration are the most frequent adverse effects [72]. It is also well known that the KD induces severe dehydration and numerous electrolyte imbalances [73]. It is therefore important to note that patients undergoing combined therapies may experience an exacerbation of dehydration and electrolyte imbalances due to the negative pharmacodynamic interaction between the two treatments. These side effects should be closely monitored.

Hyperglycaemia is a commonly recognised negative consequence of diuretic medication. Hydrochlorothiazide and furosemide reduce the rate of glucose transfer in adipose tissue [74]. Hence, similar to beta-blockers, it is imperative to note that rigorous monitoring is required when administering thiazide diuretics to patients on a KD, as their pharmacodynamics have the potential to disrupt the ketogenic state.

## 5. KD versus Haematological Agents

There is some concern that the KD might interfere with anticoagulant and antiplatelet drugs, but the number of studies exploring these interactions is limited. It has been hypothesised that the drop in INRs could be caused by an increase in levels of serum albumin as well as cytochrome P450 activity, which could be achieved by increasing dietary protein intake. It seems that the most probable explanation is a spike in warfarin metabolism caused by cytochrome P450 activation, based on the available data that show changes in drug metabolism when dietary protein consumption is increased [75].

Warfarin acts through the inhibition of vitamin K epoxide reductase (VKORC1), an enzyme that facilitates the reutilisation of vitamin K subsequent to its participation in coagulation factor carboxylation. Vitamin K undergoes a fundamental transformation from its hydroquinone state to a form that aids in the carboxylation of coagulation factors; VKORC1 then facilitates the process of converting vitamin K backwards to its active form. Vitamin K restitution is impeded by warfarin via inhibition of VKORC1, resulting in a depletion of vitamin K supplies. The combination between warfarin and vitamin K is a clinically pertinent concern [76]. Clinicians should regularly check vitamin K consumption in individuals administered warfarin. INR and PT are laboratory tests used to monitor the blood clotting time, rate, and anticoagulant medication efficiency. Patients on warfarin typically need to adhere to a modified vitamin K consumption plan. Consuming consistent daily amounts of foods with low levels of vitamin K may be important for preserving steady and appropriate INR levels [77].

Rich sources of vitamin K include green leafy vegetables such as kale, spinach, and broccoli, as well as moderate use of olive and canola oils. Animal food rich in vitamin K include chicken liver, egg yolks, hard cheeses, chicken, bacon, and gammon. Fermented foods, especially natto, provide a significant amount of vitamin K2 [78]. Each of these products is permitted on the KD [79]. As a result, warfarin-type anticoagulants should be administered to patients on a KD while the diet plan and vitamin K levels in the blood are closely monitored.

## 6. KD versus Anti-Inflammatory Agents

KD has garnered considerable attention in recent years due to the possibility that it possesses anti-inflammatory properties. Several potential mechanisms may account for the observed effects: reduced production of amyloid precursor protein [80], stimulation of PPAR-γ activation [80,81], and ketone body-induced activation of hydroxy-carboxylic acid receptor 2 (HAC2), which subsequently inhibits nuclear factor kappa B and increases prostaglandin production [82,83,84,85,86].

Each of these mechanisms has the potential to reduce inflammation in a synergistic manner, which could lead to the development of a novel strategy for treating inflammatory diseases that are resistant to conventional treatments. Additionally, this dietary approach may pave the way for novel opportunities to decrease medication dependence among chronically ailing patients and improve the efficacy of anti-inflammatory drugs by possibly allowing for lower doses of medication and reducing the risk of side effects. Furthermore, the KD selectively targets a multitude of cellular and molecular pathways that modulate inflammation in an alternative manner to conventional anti-inflammatory drug treatments [87].

### Steroidal Anti-Inflammatory Drugs

When attempting to implement a KD for patients undergoing chronic corticosteroid therapy, caution should be advised. The primary detrimental effects of corticosteroids are an increase in blood glucose levels (via stimulation of gluconeogenesis in the liver), a reduction in glucose utilisation in adipose and muscle tissue, and a decrease in insulin sensitivity [88]. These effects have the potential to disrupt nutritional ketosis. An additional prevalent adverse consequence of prolonged corticosteroid treatment is the accumulation of sodium and water [89]. When used in conjunction with the KD, the potential for hydro-electrolyte imbalances is heightened.

Hypercortisolism symptoms and hypothalamic–pituitary–adrenal (HPA) axis suppression are both known side effects of systemic corticosteroids [90]. In contrast, research conducted in the past ten years has demonstrated that the KD disrupts the hormonal equilibrium by influencing the synthesis of cortisol and other metabolic regulating hormones [91]. Actually, it was found that rats’ blood cortisol levels rise during a KD. Research conducted by Ryan et al. showed that the HPA axis may be acutely and persistently activated by a dietary treatment that involved a relative reduction of carbs, leading to nutritional ketosis [92].

## 7. KD versus CNS Disorders

### 7.1. Antiepileptic Drugs

Antiepileptic medications (AED) are commonly employed in the management of epilepsy, a prevalent neurological disorder. Nevertheless, a notable 30% of individuals suffer from refractory epilepsy, indicating their inability to attain long-lasting seizure relief despite attempting two distinct antiepileptic treatment regimens. A subset of these individuals do not meet the criteria for surgical intervention, hence requiring the exploration of alternate therapeutic approaches such as palliative surgery, neuromodulation, and adherence to a KD [93]. AEDs are categorised into two distinct classifications: liver enzyme-inducing antiepileptic drugs (EIAEDs) (phenytoin, phenobarbital, and carbamazepine) and non-enzyme-inducing antiepileptic drugs (NEIAEDs) (levetiracetam, valproate sodium, topiramate, clobazam, clonazepam, ethosuximide, gabapentin, lacosamide, lamotrigine, pregabalin, tiagabine, vigabatrin, and zonisamide) [94,95].

Considerable discussion has developed within this context regarding the potential interactions that may arise between KD and AEDs, with a specific emphasis on the ramifications for the effectiveness and safety of refractory epilepsy management [96,97,98,99,100,101].

**Figure 4:**
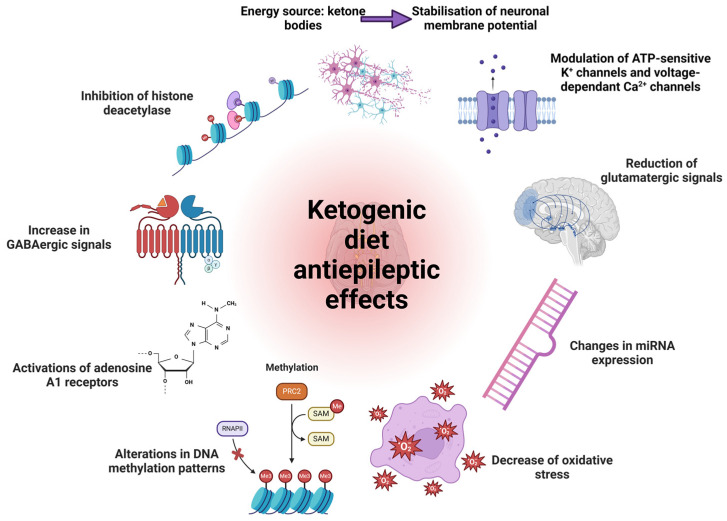
Proposed mechanisms underlying the antiepileptic effect of the KD (adapted from [93,97,104]) (created with Biorender.com) (accessed on 13 March 2024).

In recent years, the KD has been increasingly utilised as an adjunctive treatment for epileptic disorders. Numerous studies have examined the antiepileptic potential of this diet, delving extensively into the mechanisms by which nutritional ketosis produces its therapeutic effect [24,93,102,103]. The primary hypothesised mechanisms underlying the antiepileptic effect of the KD are summarised in Figure 4. Regarding the pharmacodynamic interaction between the KD and antiepileptic drugs, it is possible to discuss a synergistic effect that is either additive or potentiating, contingent upon the specific drug selected for pharmacotherapy. Figure 4 illustrates the proposed mechanisms underlying the antiepileptic effect of the KD.

With regard to pharmacokinetic interactions, nevertheless, serious caution is advised. Certain AEDs and KD, due to their high lipid content, may interact with one another in terms of absorption. Tiagabine, valproic acid, phenobarbital, and topiramate were reported to have a decreased absorption of fats, leading to reduced peak serum concentrations. Conversely, a meal rich in fat was observed to increase the rate at which phenytoin is absorbed. Moreover, soy-based foods have the potential to substantially reduce plasma concentrations of valproic acid through the facilitation of glucuronidation, thereby promoting increased rates of valproic acid excretion and clearance [105]. In contrast, consuming rufinamide with food increases peak exposure by 50% and AUC by 30–40%; thus, administering rufinamide with meals is suggested. Gabapentin should also be taken with food because it absorbs more quickly and completely when combined with a high-fat meal [95].

According to a number of studies, the KD might increase the activity of certain cytochrome P450 enzymes [106,107]. This is especially critical for antiepileptic drugs that are metabolised by these enzymes, as an increased metabolism could potentially lead to decreased plasma concentrations of the drugs and a subsequent decline in their therapeutic effectiveness [108]. A patient who initiated a KD while concurrently taking clobazam demonstrated a reduction of 42% in clobazam concentrations in their serum. The authors suggested that the KD might elevate the activity of cytochrome P450 enzymes, thereby resulting in an increased rate of drug metabolism [109]. The effect of the Atkins diet on serum concentrations of anticonvulsant medications in 63 adult patients with drug-resistant epilepsy was the subject of another study. During the trial, the following medications were administered: lamotrigine, topiramate, valproic acid, carbamazepine, clobazam, levetiracetam, lacosamide, zonisamide, and oxcarbazepine. Significant reductions in serum concentrations of clobazam, carbamazepine, and valproic acid occur after 4–12 weeks of following the modified Atkins diet. In contrast to lamotrigine, topiramate, and lacosamide, which all reduce the serum concentration, oxcarbazepine, zonisamide, and levetiracetam do not. Plasma concentrations of anticonvulsant medications may be decreased by KD, resulting in diminished efficacy and potential adverse effects [101,110,111].

### 7.2. Antipsychotic Agents

Psychotic disorders are a group of mental illnesses that include severe disturbances of perception, thinking, and behaviour, frequently leading to a loss of contact with reality. These disorders include illnesses such as schizophrenia, schizoaffective disorder, and delusional disorder. Each disorder has its own distinct symptoms and course [112]. Hallucinations and delusions are fundamental components of psychotic disorders. They can occur in several ways, such as auditory, visual, tactile, and olfactory hallucinations. In addition, they may include the maintenance of incorrect, unchanging ideas uninfluenced by contradictory data. The cause of psychotic illness is complex, with many elements at play, including genetic predispositions, neurobiological abnormalities, environmental stress, and psychosocial factors. Pharmacotherapy, particularly antipsychotic drugs, is the mainstay of treatment. However, other therapies, including psychotherapy, psychological support, and new approaches such as KD, are also receiving increased attention [113].

The association between KD and antipsychotic agents represents a significant divergence in treatment approaches for mental illness. Antipsychotic drugs have been the cornerstone of psychiatric treatment for conditions such as schizophrenia, bipolar disorder, and schizoaffective disorder, aiming to relieve symptoms such as hallucinations, delusions, and mood disorders. However, their effectiveness is often limited, with a substantial proportion of patients experiencing only partial relief of symptoms or significant side effects such as weight gain, metabolic disorders, and extrapyramidal symptoms. Instead, KD offers a new therapeutic pathway, focusing on altering the metabolic state of the brain by inducing ketosis, which involves using ketone bodies as an alternative source of energy instead of glucose. This metabolic change can influence various neurochemical pathways involved in mental illness, potentially leading to improvements in mood stability and psychotic symptoms. Furthermore, KD has shown promise for alleviating some of the metabolic side effects associated with antipsychotic drugs, such as weight gain and insulin resistance [114,115].

Regarding the therapeutic management of schizophrenia, it is mainly represented by antipsychotic drugs targeting dopamine activity. The KD has attracted attention because of its potential impact on brain function. Ketosis can alter levels of neurotransmitters, particularly GABA and glutamate, which play a crucial role in the pathology of schizophrenia. Studies suggest that ketogenic diet-induced ketosis may increase GABA synthesis while modulating glutamate metabolism, which may improve symptoms associated with schizophrenia. However, there are challenges, including adherence to dietary restrictions and potential adverse effects on lipid profiles and metabolic health. Nevertheless, emerging evidence suggests that ketogenic diet therapy holds promise as an adjunctive treatment for schizophrenia, offering a novel approach to addressing treatment-resistant symptoms and improving overall patient outcomes [114]. A study on mice showed the effects of co-administration of the KD with the antipsychotic drug olanzapine. It has been shown that the KD can exert synergistic effects with antipsychotic drugs by modulating neurotransmitter systems, stabilising neuronal networks, and improving neuroplasticity. The adverse effects of olanzapine, blamed for decreased insulin secretion and insulin resistance, were neutralised by diet-induced ketosis. Compared to the control and control groups, an enhanced therapeutic effect and greater safety were demonstrated when olanzapine was administered to mice in the ketosis state [116].

In a retrospective study of 31 patients undergoing antipsychotic treatment, researchers demonstrated the need to reduce the dose of the drug or even eliminate the antipsychotic from therapy in patients who followed a ketogenic diet [113]. The diet also showed significant effects in two cases of treatment-resistant schizophrenia. Patients treated concomitantly with lithium, olanzapine, ziprasidone, aripiprazole, lamotrigine, quetiapine, haloperidol, perphenazine, and risperidone stopped taking medication after several months. They continued to follow the diet, and the hallucinations and suicidal thoughts disappeared. Their mood improved significantly, and they became independent without needing specialist care or medication [117].

In children with autism spectrum disorders, IQ scores were found to increase when the KD was combined with drug therapy [118].

A negative aspect of the concomitant administration of antipsychotic medication in patients with the KD is the accentuation of metabolic adverse reactions such as constipation, fatigue, acidosis, and dehydration. In patients with autism spectrum disorders or bipolar disorder, these reactions are an impediment to continuing the diet, especially for children [115].

### 7.3. Anxiolytics and Hypnotic Agents

Anxiolytics and hypnotic agents (benzodiazepines, zolpidem, zaleplon, eszopiclone, etc.) are commonly prescribed medications for the management of anxiety disorders and insomnia. There is no evidence in the literature that these medications and the KD interact directly. However, we can highlight potential indirect interactions based on their pharmacological properties and the metabolic effects of the KD.

The KD may affect the activity of liver enzymes involved in drug metabolism, such as cytochrome P450 enzymes. Anxiolytics and hypnotic agents are metabolised in the liver, which means that diet-induced changes in CYP P450 activity could theoretically affect their metabolism and elimination from the body. This may lead to changes in the effectiveness of the drugs or adverse effects [105].

In addition, the ketogenic diet influences neurotransmitter levels and neuronal excitability in the brain, which could interact with the mechanisms of action of anxiolytic and hypnotic agents. For example, benzodiazepines enhance the inhibitory effects of gamma-aminobutyric acid (GABA) in the central nervous system, leading to sedative and anxiolytic effects [119].

### 7.4. Antidepressants

Regarding the choice of a ketogenic diet in patients on antidepressant medication, no information has been found to show a direct link between the two. According to the observations so far from clinical trials, it can be assumed that there is a synergistic therapeutic effect. Both the ketogenic diet and selective serotonin reuptake inhibitors (SSRIs) or serotonin and norepinephrine reuptake inhibitors (SNRIs) have a significant impact on the neurotransmitter systems and metabolic pathways of the brain on their own. SSRIs influence mood and emotional control by blocking serotonin reuptake, which increases synaptic serotonin levels. On the other hand, SNRIs influence the reuptake of both norepinephrine and serotonin, which affects both neurotransmitter systems. The high-fat, low-carb ketogenic diet causes the body to change its metabolism to one that uses ketone bodies, which affects neurotransmitter creation and neuronal energy consumption [120].

### 7.5. General Anaesthetics

The ketosis state characteristic of the KD leads to high levels of ketone bodies, which serve as an alternative energy substrate for the brain. This “fuel” can potentially influence the body’s response to general anaesthesia, which usually involves the administration of anaesthetic agents that modulate neurotransmitter systems and neuronal activity. In addition to direct interactions as a result of the ketosis state, the administration of general anaesthetics, such as sevoflurane, in patients undergoing the KD determines possible implications for anaesthetic depth, pharmacokinetics, and recovery dynamics. In addition, the impact of the KD on systemic metabolism, electrolyte balance, and liver function could influence the metabolism, distribution, and elimination of anaesthetic drugs [121].

A condition of ketosis brought on by a high-fat, low-carb diet may have anticonvulsant benefits via boosting neuronal energy stores, antioxidant capabilities, and anti-inflammatory effects [122].

The effects of the KD on serum electrolytes and acid–base status, the impact of intravenous fluid selection on acid–base status, the modification of ketogenic status through the administration of glucose in intravenous fluids or medication, the risk of hypoglycaemia, and the effects of ketosis and acidosis on cardiovascular function are among the specific perioperative concerns of patients undergoing the KD [30].

The acidosis caused by the KD is usually not severe and has little effect on perioperative care or physiological function. On the other hand, kidney stones, decreased bone mineralisation, and changes in blood electrolyte levels might all be long-term consequences of chronic acidosis [123,124].

Severe acidosis can occasionally be observed after extended surgical procedures or when the patient is subjected to additional stressors, such as dilutional acidosis from non-buffered intravenous fluids, metformin, or medications that inhibit carbonic anhydrase (zonisamide or topiramate) [125].

Regarding the use of propofol in general anaesthesia, propofol-related infusion syndrome has been reported in children following the KD, a very rare but potentially fatal reaction [126].

### 7.6. Cannabidiol (CBD)

CBD is the non-psychoactive component of cannabis, which, although controversial, is associated with various potential health benefits, including relief of pain, anxiety, inflammation, and seizures. While there is little direct research on the interaction between the KD and CBD specifically, there are some indirect ways in which the two might interact [99,127].

Both the KD and CBD influence metabolism. The KD alters metabolism to prioritise fat burning for energy, while CBD may affect metabolism through its interaction with the endocannabinoid system. However, there is no clear evidence to suggest that the KD would significantly alter CBD metabolism.

Because the KD is high in fat, it could enhance CBD absorption, as CBD is a fat-soluble compound. Consumption of CBD with high-fat foods or in the context of a high-fat diet could lead to better absorption and potentially stronger effects.

Compared to their beneficial effects in the management of conditions such as epilepsy or chronic pain, we can consider the existence of a synergistic effect between the two. Both have been studied independently for their potential neuroprotective and anti-inflammatory properties, so combining them could theoretically enhance their effects in certain situations.

Both the KD and CBD can have side effects such as digestive problems, appetite changes, and mood swings. Combining the two could exacerbate these side effects for some people, although this would likely vary depending on the individual and their specific health condition [127]. The KD can also induce overexpression of cannabidiol receptors, resulting in a therapeutic underdose of CBD [128].

## 8. KD versus Cancer

According to the latest studies in the field, the KD may be an adjuvant therapy in the advanced treatment of various cancers. Acidosis and lowering the concentration of available glucose may prevent the growth of cancer cells. Combining the KD with classical cancer therapy leads to decreased tumour cell growth [129,130,131,132].

The synergism of using the KD concurrently with chemotherapy has been demonstrated in several cancers. In a study of 518 women with recurrent or metastatic local breast cancer, the effect of irinotecan in combination with the KD was studied. Patients in the study were randomly assigned to the combination intervention group or the control group, followed by treatment with irinotecan + ketogenic diet or irinotecan + normal diet, respectively. Irinotecan sensitivity, response rate to therapy, survival, quality of life, incidence of adverse reactions, and cost-effectiveness were followed. A longer response time was observed with irinotecan administration in patients with a normal diet [133].

Another study demonstrated the effectiveness of the KD in pancreatic cancer. The KD has made phosphoinositide 3-kinase inhibitors (PI3K) inhibitors, which are normally inactive against pancreatic cancer, effective in KPC tumours. Here, we will show that in the model KPC mouse (KrasG12D/+; Trp53R172H/+; P48-Cre), the ketogenic diet synergizes with the clinically active cytotoxic chemotherapy regime of gemcitabine, nab-paclitaxel, and cisplatin. Surprisingly, while it has no effect on tumour growth on its own, the ketogenic diet triples the survival benefits of triple chemotherapy [134].

Also, another study demonstrated increased therapeutic efficacy and decreased incidence of adverse reactions for oxaliplatin and leucoplatin in patients diagnosed with stage III-IV locally advanced or metastatic gastric adenocarcinoma that received metabolically supported chemotherapy and followed a KD [135].

A study in a murine model of lung cancer revealed the metabolic effects of radiotherapy-associated KD. Different concentrations of glucose and beta-hydroxybutyrate (bHB) were administered to simulate different levels of ketone bodies, combined with radiotherapy, on LLC cell proliferation. The KD may enhance the anti-tumour effect of radiotherapy in LLC tumour-bearing mice by reducing glucose and increasing the energy supply ratio from fat [136].

The KETOCOMP study highlighted the impact of KD on the body composition of patients with different types of cancer during radiotherapy. After initial water losses, KD tends to reduce body weight and fat mass while maintaining fat-free muscle mass and skeletal mass, which are essential in breast cancer [137]. In head and neck cancer, combining the KD with radiotherapy had beneficial effects compared to chemotherapy [138].

## 9. KD on the Microbiome

The KD has been seen to cause substantial alterations in the makeup of the gut microbiota. These changes are marked by a decrease in diversity and shifts in the relative abundance of certain bacteria. The effects of probiotics and microbiota-modifying medications are exerted via direct influence on the structure and function of the gut microbial population. Preclinical studies have shown that there may be interactions between the KD and some probiotics. These interactions may have either a synergistic impact, where the two work together to improve metabolic health, reduce inflammation, and enhance gastrointestinal function, or an antagonistic effect, where they work against each other. Probiotic treatment may reduce the negative effects of KD-induced dysbiosis and relieve gastrointestinal symptoms that are often linked to KD, such as constipation and dyspepsia. On the other hand, medications that change the makeup of the gut microbiota, including antibiotics or prebiotics, might disturb the metabolic effects of the KD and weaken its effectiveness as a treatment [139].

In an animal model of childhood epilepsy, how the KD affects hepatic steatosis and its modulation by a defined probiotic mixture was studied. An assessment was conducted using liver homogenates to measure several factors, including malondialdehyde levels, fatty acid profiles, mRNA expression of enzymes involved in lipid metabolism, mitochondrial function, histone deacetylase activity, cytokines, and chemokines. The liver homogenates were used to analyse a combination of several measures, including malondialdehyde levels, fatty acid profiles, mRNA expression of enzymes associated with lipid metabolism, mitochondrial function, histone deacetylase activity, as well as cytokines and chemokines [140].

The outcomes of the study showed that the use of the KD led to a decrease in seizures, but it also caused severe hepatic steatosis. This condition is characterised by a white liver, a buildup of triglycerides, increased levels of malondialdehyde, and decreased levels of polyunsaturated fatty acids and acyl-carnitines compared to yearlings that were given a normal diet. The metabolic phenotype caused by the KD was averted by simultaneously administering a combination of *Streptococcus thermophilus* HA-110 and *Lactococcus lactis subsp. lactis* HA-136. The probiotic combination provided liver protection by enhancing pAMPK-mediated signalling and stimulating lipid oxidation. The strains further increased the production of caspase 1 and interleukin 18, perhaps contributing to their hepatoprotective impact in this animal. These findings indicate that administering probiotics at an early stage might be a potential strategy to mitigate the risk of liver-related complications in children who follow a medically prescribed ketogenic diet [140,141].

## 10. Other Pharmacotherapeutic Interactions

### 10.1. Pharmacodynamics of Ketoacidosis-Inducing Agents

Nutritional ketosis is a flexible metabolic state that has several therapeutic implications, namely, in the areas of metabolic health and the treatment of various diseases. Ketosis is characterised by elevated levels of blood ketone substances, including acetoacetate, beta-hydroxybutyrate, and acetone. These groups of molecules are produced when there is an increase in the oxidation of fatty acids or when there is a reduced carbohydrate intake, such as when following a KD. Fatty acids are the metabolic precursors of ketone molecules. Acetoacetate and beta-hydroxybutyrate ketone bodies serve as alternate energy sources in peripheral tissues, while acetone is eliminated by the lungs and urine [17,142,143,144,145]. 

On the other hand, diabetic ketoacidosis (DKA) is a serious metabolic complication of diabetes that is characterised by high blood sugar levels, elevated ketone levels, abnormalities in electrolytes, increased osmolarity, and metabolic acidosis [146]. This condition is a result of insufficient insulin and the increase in chemicals that lead to insulin resistance, including glucagon, growth hormone, and catecholamines. Diabetic ketoacidosis is most frequently caused by a decrease in insulin activity or an increase in insulin demand. This can happen as a result of missed doses, incorrect delivery, or infections. This results in the inability to transfer glucose into the cells, causing cellular malnourishment and starvation. The majority of cells transition to using free fatty acids as their primary source of energy. In the absence of insulin, there is an abundance of free fatty acids in the circulatory system, which are carried to the liver and then transferred through the mitochondria for the process of oxidation [147,148,149]. Inadequate insulin levels lead to excessive ketone generation [150]. DKA is more prevalent in young children and adolescents compared to adults, although it can manifest at any age [151].

Simultaneously, it is widely recognised that there is a diverse range of medicines that can have the adverse effect of triggering diabetic ketoacidosis [152]. Table 1 displays the primary categories of medications frequently used in pharmacotherapy that have the potential to induce diabetic ketoacidosis.

Thus, the use of the KD at the same time with various drugs that can induce or aggravate diabetic ketoacidosis may increase the risk of serious metabolic complications, through a pharmacodynamic adverse effect of synergistic potentiation of ketone body formation. For this reason, clinicians should carefully monitor pharmacotherapy as well as dietary therapy, which emphasises the importance of individualised and patient-centred pharmacotherapy.

### 10.2. Pharmacokinetics of Lipophilic Drugs

Numerous studies have demonstrated that a high-lipid diet can enhance the absorption of lipophilic medications [190,191,192,193,194]. Petit et al. discovered that a sustained high-fat diet alters gastrointestinal physiology and the manner in which the body utilises lipids, resulting in enhanced lipid absorption capacity [190]. Furthermore, Patel and Brocks noted that pharmaceuticals that possess a significant level of lipophilicity may demonstrate elevated bioavailability when administered in conjunction with diets high in fat [191]. There are a number of ways in which dietary lipids and lipid-based formulations could affect the oral absorption of lipophilic medications. By forming different types of colloidal structures, the drug’s solubility can be enhanced. The presence of lipids and the modelling of physiological lipid processing pathways can affect drug solubilisation by increasing the release of bile salts and phospholipids [193]. Some of the most widely used medications with high lipophilicity are shown in Table 2.

The impact of a high-fat diet on the absorption of lipophilic drugs may result in fluctuations in drug effectiveness and an elevated likelihood of adverse effects, given that heightened drug absorption corresponds to an intensified drug mechanism and the consequent danger of toxicity. When prescribing lipophilic drugs, clinicians should consider the patient’s dietary habits and potentially modify dosages in order to maintain therapeutic levels and minimise side effects. This approach ensures patient safety and optimises treatment outcomes.

### 10.3. Drugs Disrupting Ketosis

In order for the KD to reach its full therapeutic potential, it is imperative that the state of nutritional ketosis be maintained throughout the course of treatment. However, the state of ketosis can be disrupted by the consumption of carbohydrates [220]. While therapeutic guidelines and clinicians diligently monitor patients to advise them against consuming carbohydrate-rich foods, particular emphasis should be placed on pharmaceutical formulations that incorporate carbohydrates either as active ingredients or excipients.

Lactulose, also known as 4-O-β-D-galactopyranosyl-D-fructofuranose, is a widely recognised carbohydrate that is frequently employed in the management of chronic constipation [221]. Inulin, an increasingly prevalent prebiotic in the nutraceutical industry, is a carbohydrate derivative composed of two to sixty fructose units connected to a terminal glucose by β-(2, 1) glycosidic bonds [222].

Simultaneously, the pharmaceutical industry extensively utilises carbohydrates as excipients in pharmaceutical technology [223]. The primary categories of carbohydrates commonly utilised in both liquid pharmaceutical forms (such as syrups, solutions, injectable or infusible solutions) and solid pharmaceutical forms (such as tablets and capsules with regular or modified release) include sugars like sucrose, glucose, lactose, mannitol, and sorbitol, which are employed as sweeteners [224]. Additionally, cellulose-type polysaccharides or cellulose derivatives (such as microcrystalline cellulose, hydroxypropyl methylcellulose, and carboxymethylcellulose) are primarily used as thickening agents [225]. Starch-type polysaccharides serve as disintegrating agents, binding agents, and filling agents [226]. Dextran-type polysaccharides are used as stabilising agents for parenteral solutions [227]. Various types of gums (such as xanthan, acacia, and guar) are employed as emulsifying agents or thickening agents [228]. Lastly, cyclodextrins are used to enhance the absorption of different classes of active substances [229].

A further investigation determined that anticonvulsant, sedative, and antibiotic medications include significant quantities of propylene glycol and other carbohydrates. These substances have the potential to disrupt nutritional ketosis and exacerbate propylene glycol poisoning and metabolic acidosis [230].

Hence, to enhance the therapeutic benefits of the KD, it is crucial to take into account the existence of various categories of carbohydrates in pharmaceutical formulations.

## 11. Identified Research Gaps and Future Perspectives

The findings derived from this narrative review reveal some potential future prospects with therapeutic consequences, along with several gaps in the existing research. There is a need for a more comprehensive investigation of the potential processes that may contribute to drug interactions with KD. While the impact of the KD on the pharmacokinetics of certain medications has been identified, there are still certain mechanisms that have not been fully understood. Subsequent investigations should prioritise understanding of these pathways at the molecular scale to enhance the precision of forecasting potential KD-drug interactions.

Additionally, clinical trials examining the long-term effects of combining the ketogenic diet with various medications used to treat chronic diseases are required to assess its effectiveness and safety. Concurrently, the primary aim of this investigation ought to be the development of clear, extensively documented protocols that apply to a broad spectrum of health practitioners. In consideration of the inter-individual variability of each patient, these recommendations may facilitate the tailoring of therapeutics via dietary and treatment plans.

Nevertheless, upon conducting a comprehensive review of the existing literature, several deficiencies in the research could be identified. One of these challenges is the absence of study standardisation, as substantial discrepancies exist among studies concerning the implementation of the KD. This characteristic limits the ability to compare studies and derive conclusive findings. Additionally, the literature fails to adequately represent certain patient populations, including children, pregnant women, the elderly, and patients with special needs. Changes in pharmacokinetic parameters and other physiological modifications that occur in these patient populations are crucial to comprehend how the KD influences drug mechanisms and metabolism. However, it is important to note that the scientific literature contains a lack of research examining the effects of dietary associations in various pathologies on patients’ mental health, general well-being, quality of life, and satisfaction.

The present narrative review conducted a thorough and focused investigation into the dual function of KD as a pharmacotherapeutic potentiating agent, exhibiting both positive and negative effects. In addition, a diverse range of chronic pathologies and pharmacotherapeutic regimens are provided, thereby offering a holistic approach to a subject that is rarely addressed in the scientific literature. Through this approach, the present study offers numerous clinically applicable insights regarding the incorporation of the KD into conventional pharmacotherapeutic regimens.

## 12. Conclusions

This narrative review has offered various perspectives on the prospective benefits of incorporating the KD into the treatment of chronic pathologies. While KD has demonstrated encouraging results in treating various clinical conditions (e.g., diabetes, epilepsy, and cardiovascular diseases), these results must be assessed in a cautious and balanced manner in order to comprehend the potential negative consequences that may arise from inadequate monitoring of clinical outcomes. In addition, it is important to fully understand the adverse effects of KD and its potential interactions with multiple medication classes to improve the patients’ adherence to treatment and their quality of life. To ensure the efficacy of this nutritional intervention, it should be emphasised that the diligent selection of patients who qualify for KD implementation and their careful monitoring throughout treatment, particularly patients with renal and hepatic dysfunction, are crucial. Consequently, although KD presents numerous and highly beneficial opportunities for integration with pharmacotherapeutic regimens, this integration must be performed exclusively by specialists (physicians, pharmacists, and nutritionists) to ensure that patients receive a personalised therapy that is both safe and effective.

## Figures and Tables

**Table 1:** Ketoacidosis-inducing drugs.

ATC	Therapeutics	Chemical/Pharmacological Class	Compounds	Observations	Reference
A10	DRUGS USED IN DIABETES	Insulin and analogues	Insulin	Improper administration or incorrect handling	[153]
		Sodium-Glucose Cotransporter-2 (SGLT2) Inhibitors	Canagliflozin, dapagliflozin, empagliflozin	Because of their ability to promote increased breakdown of fats and elevated levels of glucagon	[154,155,156]
C01	CARDIAC THERAPY	Sympathomimetics	Epinephrine, norepinephrine, terbutaline		[157,158,159]
C02DA	DIURETICS	Thiazides	Hydrochlorothiazide, chlorthalidone		[160,161,162]
H02	CORTICOSTEROIDS FOR SYSTEMIC USE	Glucocorticosteroids	Prednisone, dexamethasone	At high concentrations, such as those used to alleviate intracranial tumours	[152,163,164]
J05	ANTIVIRALS FOR SYSTEMIC USE	Integrase Strand Transfer Inhibitor (INSTI)	Raltegravir, elvitegravir, dolutegravir	The usage of INSTI was linked to a higher risk of developing new-onset diabetes mellitus or hyperglycaemia within the first 6 months after starting antiretroviral therapy	[165,166,167]
J05		HIV Protease inhibitors	Ritonavir		[168]
L01	ANTINEOPLASTIC AGENTS	Checkpoint Inhibitors	Pembrolizumab, nivolumab, ipilimumab		[169,170,171]
		Chemotherapy drugs	L-asparaginase		[172,173,174]
L03	IMMUNOSTIMULANTS	Interferons	Interferon alpha		[175,176]
L04	IMMUNOSUPPRESSANTS	Calcineurin inhibitors	Tacrolimus	Immunosuppressive medicines administered post-transplantation are a primary risk factors for diabetic ketoacidosis.	[177,178]
N02B	ANALGESICS AND ANTIPYRETICS	Salicylates	Salicylic acid derivates	High anion gap acidosis is a common symptom of paediatric overdose, whereas adults may experience a combination of respiratory alkalosis and metabolic acidosis	[179]
N03	ANTIEPILEPTICS	Anticonvulsivants	Valproate, phenytoin		[180,181,182]
N05A	ANTIPSYCHOTICS	Atypical Antipsychotics	Clozapine, olanzapine	DKA can manifest suddenly and without weight increase	[183,184]
		Mood stabilisers	Lithium		[185,186]
R03A	ADRENERGICS, INHALANTS	Beta-adrenergic agonists	Albuterol, salmeterol	Although insulin secretion is enhanced due to specific beta(2)-agonist actions on pancreatic beta cells, overall serum glucose levels are raised and insulin sensitivity appears to be decreased due to other mechanisms, such as increased glucagon production and hepatic effects	[187,188,189]

**Table 2:** Medications with a high lipophilicity.

Chemical/Pharmacological Class	Compounds	Reference
Antipsychotics	Olanzapine, clozapine	[195]
Antidepressants	Amitriptyline, nortriptyline, doxepin	[196,197,198]
Benzodiazepines	Diazepam, midazolam	[199]
Sedatives	Zolpidem, zopiclone	[200,201]
Antiepileptics	Phenytoin, carbamazepine, valproic acid, gabapentin, pregabalin	[202,203]
Antiarrhythmic drugs	Amiodarone	[204]
Beta-blocking agents	Propranolol, metoprolol	[205]
Statins	Simvastatin, fluvastatin, lovastatin, pitavastatin, and atorvastatin	[206]
Antimalarian drugs	Chloroquine, mefloquine	[207,208]
Antifungal drugs	Ketoconazole, itraconazole	[209,210]
Immunosuppressants	Tacrolimus	[211]
Antivirals	Ritonavir, saquinavir	[212,213]
Opioids	Methadone	[214]
Antihistamines	Cetirizine, loratadine	[215,216]
Antiparasitic drugs	Ivermectin	[217]
Antituberculosis	Rifampicin	[218]
Diuretics	Spironolactone	[219]
